# Suitable Habitats of Two Tea Pests for Management Guidance in China Under Climate Change

**DOI:** 10.3390/insects17070740

**Published:** 2026-07-20

**Authors:** Zhengxue Zhao, Xueli Feng, Jing Zhou, Maoyuan Yao, Jianxin Chen, Xiudong Huang

**Affiliations:** 1 College of Agriculture, Anshun University, Anshun 561000, China; zzx611324@163.com (Z.Z.); maoyuanyao723@163.com (M.Y.); jianxinchen000@163.com (J.C.); 2 Department of Energy Engineering, Guizhou Technological College of Ecology and Energy, Guiyang 550003, China; 15885378036@163.com; 3 Anshun Academy of Agricultural Sciences, Anshun 561000, China; hxdmx1213@163.com

**Keywords:** climate change, distribution prediction, pest management, species distribution model, tea pests

## Abstract

Driven by the human demand for tea beverages, tea production has expanded worldwide. Tea production in China, the world’s largest tea producer, is constrained by *Dendrothrips minowai* and *Matsumurasca (Matsumurasca) onukii*. Various management measures for controlling these pests have been developed, but their implementation requires knowledge of the pest distribution, which is currently insufficient. Therefore, precise management of these pests is a major challenge. Using optimized MaxEnt models for the distributions of the two pests across the current and future timeframes, we predicted the overlap of their suitable habitats. The central and southern provinces of China were identified as the primary suitable habitats of both pests at the current time. The suitable habitats will diverge in the future, with *D. minowai* habitats declining by 29.70–61.90% and *M. onukii* habitats increasing by 8.05–43.62%. These results demonstrate species-specific responses to climate change. Despite a decrease in overlap areas, the current and future overlap areas consistently coincide with some major tea-growing areas such as Guizhou, Yunnan, Hunan, and Fujian. The predicted overlap areas can aid the identification of priority areas, optimization of resource allocation, and dynamic adjustment of management measures, improving the precision and efficiency of managing the two pests.

## Simple Summary

*Dendrothrips minowai* and *Matsumurasca (Matsumurasca) onukii* are two major tea pests in China that seriously threaten the tea industry. *D. minowai* has rasping–sucking mouthparts and forewings fringed with long hairs, while *M. onukii* has piercing–sucking mouthparts and forewings not fringed with long hairs. To achieve precise management of these two pests, we predicted their suitable habitats under current and future environmental conditions. The predictions showed that the current suitable habitats of both pests are heterogeneous, mainly confined to the central and southern regions. Future environmental conditions will lead to a decrease in the suitable habitat of *D. minowai* and an increase in that of *M. onukii*. Furthermore, the overlap of suitable habitats for the two pests will decrease in the future; however, these habitats will still spatially coincide with some major tea-growing areas. Our predictions will contribute to the precise formulation of pest management measures.

## 1. Introduction

Tea beverages, made by steeping or boiling tea leaves in water, are the second most popular beverages, consumed by more than 3 billion people worldwide [1,2]. To satisfy such widespread demand, tea-tree (*Camellia sinensis* (L.) Kuntze) plantations have been established in many countries [3,4]. With the largest tea-tree plantation area (>60% of the world’s total; [5]), China has become the largest producer and consumer of tea and a major exporter of tea to other countries [3,6]. Consequently, the tea industry plays an appreciable role in China’s economic growth [7]. Unfortunately, frequent attacks on tea trees by multiple pest species have adversely affected this industry [8].

*Dendrothrips minowai* (Priesner, 1935) (Thysanoptera: Thripidae) and *Matsumurasca (Matsumurasca) onukii* (Matsuda, 1952) (Hemiptera: Cicadellidae) are two major pests of tea trees in China. The nymphs and adults of *D. minowai* and *M. onukii* feed on the sap of tea buds, leaves and young shoots [9,10,11]. However, the different structures of their mouthparts result in different feeding methods. Specifically, the former obtains sap through rasping [11], and the latter obtains sap through piercing [9,10]. Tea leaves damaged by *D. minowai* show linear scars, uneven surfaces, discoloration, and leaf abscission [11], while tea leaves damaged by *M. onukii* typically show chlorosis, curling, browning, and necrosis [9,10]. The ultimate decline in tea production incurs significant economic losses in the tea industry [12,13]. For instance, *M. onukii* infestation has reduced tea production by 10–15% in the middle and lower reaches of the Yangtze River. During outbreaks, the tea production loss can exceed 50% [14]. Both *D. minowai* and *M. onukii* have short development periods and can produce multiple generations per year [11,15]. The consequent rapid establishment and expansion of populations is the foundation of outbreaks. At present, *D. minowai* and *M. onukii* have spread to more than ten tea-growing provinces in China [16,17]. Research on the management of these two pests has variously focused on genetic structure [18,19], behaviors [15], and control measures [13,20,21]. Although these studies have provided a wealth of important information, precision management requires knowledge of the two pest distributions, which have been inadequately investigated. Limited knowledge of the two pest distributions is the primary challenge in current precision-control practices.

Manual survey methods have constrained our knowledge of *D. minowai* and *M. onukii* in China to several small areas [22,23]. As previously mentioned, both pests are now distributed across numerous provinces, indicating that the pests are environmentally adaptable to an extensive range of environmental conditions. Nevertheless, they remain undetected in a substantial number of China’s tea-growing areas, likely reflecting the inadequate coverage of manual surveying rather than a true absence of the pests. The actual distribution of these two pests is anticipated to extend beyond the currently observed range. To better understand the distributions of *D. minowai* and *M. onukii* throughout China, methods beyond manual surveying should be applied. In addition, previous studies have reported that *D. minowai* and *M. onukii* occupy similar environmental spaces [24], leading to a certain degree of overlap in their distribution ranges. Such overlap areas are the probable high-risk zones of co-infestation damage to tea trees. By recognizing overlap areas, farmers and plant protection organizations could establish spatial priority rankings for management and intervention, facilitating precise control and optimized allocation of resources. For instance, the release of natural enemies and application of pesticides can be prioritized in overlap areas, effectively mitigating the risk of outbreaks and thereby reducing tea production losses.

Species distribution models are key tools for investigating broad-scale species distributions, as they predict the future spatial-distribution patterns of species. Represented by MaxEnt as the mainstream model, species distribution models have been widely applied to crop protection. Future dynamic changes in pest distribution can reveal the spread trends, enabling the deployment of management measures that adapt to climate change. A previous study predicted the *M. onukii* distribution in China using the MaxEnt model [14], but the results were rendered imprecise by inappropriate modeling procedures. Furthermore, the distribution of *D. minowai* in China has not been reported. To improve this situation, we provide MaxEnt-based predictions of the *D. minowai* and *M. onukii* distributions in China. After tracking the present and future distribution patterns of suitable habitats and spatial variation over time, we explore the changes in spatial patterns in overlap areas.

## 2. Materials and Methods

### 2.1. Distribution Records

**Figure 1:**
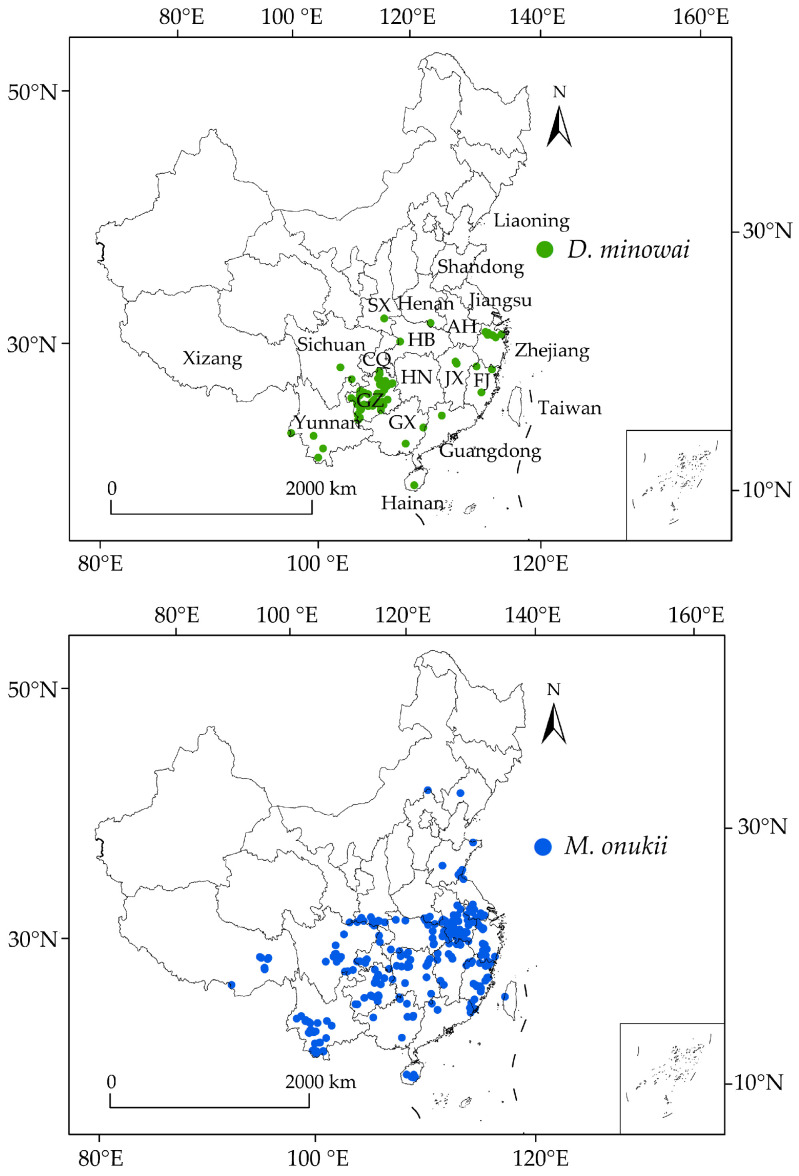
Distribution records of *D. minowai* and *M. onukii* in China. AH: Anhui, CQ; Chongqing, FJ: Fujian, HB: Hubei, HN: Hunan, GX: Guangxi, GZ: Guizhou, JX: Jiangxi, SX: Shaanxi.

A MaxEnt model requires the latitude and longitude data of the distribution records of the target species. In this study, the *D. minowai* and *M. onukii* records in China were obtained from the literature retrieved from Web of Science and China National Knowledge Infrastructure, using the Chinese and Latin names of the target species as search terms. Doubtful records were manually removed. Latitude and longitude data were used directly if present in the distribution records and queried using Google Earth if not reported. Using the Latin names, we also searched for *M. onukii* distribution records containing latitude and longitude data on the Global Biodiversity Information Facility website (https://www.gbif.org/, accessed on 19 April 2026) [25]. Subsequently, problematic records were automatically flagged using the CoordinateCleaner Package in R 4.3.3 [26]. After collecting the distribution records, spatial thinning at a 5 km distance threshold was performed using the spThin 0.2.0 package in R 4.3.3 [27,28]. Ultimately, we obtained 70 *D. minowai* and 246 *M. onukii* distribution records containing latitude and longitude data in China (Figure 1 and Table S1).

### 2.2. Current and Future Environmental Variables

The MaxEnt model also requires environmental variables. To predict the current and future suitable habitats of the two pests, we employed environmental variables for current and future timeframes. We downloaded 19 bioclimatic variables (temperature-related variables BIO1–BIO11 and precipitation-related variables BIO12–BIO19) with a resolution of 2.5 arc min from the WorldClim website (https://www.worldclim.org accessed on 13 September 2024), representing current (1970–2000) and future periods. The bioclimatic variables of the future timeframes are derived from the averages of five global climate models [28]: BCC-CSM2-MR, HadGEM3-GC31-LL, IPSL-CM6A-LR, MIROC6, and MPI-ESM1-2-HR. During this stage, we selected Shared Socioeconomic Pathways (SSPs) 126 and 585 from 2041 to 2060 (2050s) and 2061 to 2080 (2070s). We also created slope and terrain ruggedness in the terra package for R 4.3.3 [29] using elevation data (resolution: 2.5 arc min) obtained from the WorldClim website. These three datasets represented the topographic variables. We then determined the geographical distribution of the host plants (tea trees) of both pests. The availability of tea trees was represented by the tea-growing area data downloaded from the SPAM 2020 v1.0 Global dataset at 1 km [30] and then resampled to a spatial resolution of 2.5 arc min for consistency with the other variables. The future topographic variables and tea-growing area were represented by modern data, assuming that the future data will not substantially change and given the unavailability of future-specific data.

To avoid the collinearity problem, the environmental variables were screened by first extracting their values from the distribution records. Subsequently, the variable pair with the highest correlation (>0.7) was identified in the usdm package for R 4.3.3 [31] and the variable with the highest variance inflation factor (VIF) was excluded. This process was repeated until the correlation coefficients of all variable pairs were less than 0.7. We further verified that the VIF values of all retained variables were below 10, indicating no significant collinearity. Ultimately, eight environmental variables for each pest were selected and applied in the respective models.

### 2.3. MaxEnt Model

We tuned the feature combination and regularization multiplier value in the MaxEnt model. Eight feature combinations (L, LQ, LQH, HPT, QHP, LQHP, QHPT, and LQHPT) were constructed from the five feature types: Linear (L), Quadratic (Q), Product (P), Threshold (T), and Hinge (H) and tested in the ENMeval package for R 4.3.3 [32]. Meanwhile, the regularization multiplier value ranged from 0.2 to 6 in intervals of 0.2. Using the block method, this step yielded 240 candidate models for each pest. The model parameters that minimized the corrected Akaike information criterion (AICc) were selected as the optimal parameters. Finally, the QHPT and HPT feature combinations were selected for *D. minowai* and *M. onukii*, respectively, with regularization multiplier values of 1.8 and 1.2, respectively. We also reported the area under the receiver operating characteristic curve (AUC) and partial AUC (pAUC) ratios of the optimal models using the ENMeval and kuenm packages in R 4.3.3 [32,33]. Models yielding AUC values and pAUC ratios above 0.9 and 1.5, respectively, were judged as excellent performers. The final MaxEnt model of the two pests was developed in the R 4.3.3’s dismo package [34] using 5-fold cross-validation, 10,000 background points, and a cloglog output format [35]. The relative importances of the environmental variables were assessed through a permutation method that identified the key factors influencing the distributions of the two pests. Subsequently, the relationships between these key factors and pest-presence probability were observed in response curves generated by the model.

The potential extrapolation risk was assessed using multivariate environmental-similarity surfaces in the dismo package for R 4.3.3 [34,36], which quantifies the similarity between any given point and a reference set of points with respect to selected environmental variables. According to previous studies [28,37], extrapolation risk is absent if the similarity is zero or positive and present if the similarity is less than zero.

### 2.4. Visualization Analysis

Suitable and unsuitable habitats were delineated using maximum training sensitivity plus a specificity threshold that separates the suitable regions (above the threshold) from unsuitable regions (below the threshold). After running the MaxEnt model, the thresholds were determined as 0.17 for *D. minowai* and 0.28 for *M. onukii*. Furthermore, the suitable habitats of the two pests were overlaid to identify overlap areas and nonoverlap areas. The overlap areas were defined as zones where *D. minowai* and *M. onukii* co-occurred, and nonoverlap areas were defined as the sum of the zones exclusively occupied by either pest.

## 3. Results

### 3.1. Model Evaluation and Variable Contributions

**Figure 2:**
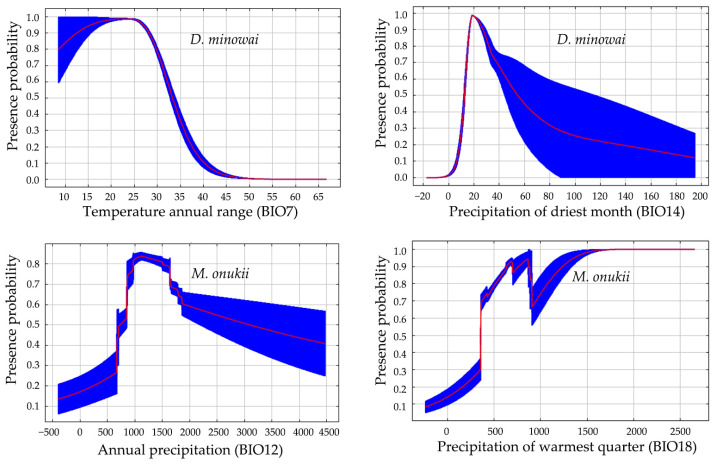
Response of pest-presence probability to two key environmental variables.

The optimized MaxEnt models for *D. minowai* and *M. onukii* demonstrated excellent performance, as indicated by their AUC values and pAUC ratios (Table 1). The environmental variables contribute unequally to the distributions of the two pests (Table 2). The distribution of *D. minowai* depends primarily on the temperature annual range (BIO7) and secondarily on the precipitation of the driest month (BIO14) (Table 2). The contributions of the remaining six variables are comparatively small (Table 2). The *M. onukii* distribution is determined mainly by annual precipitation (BIO12), with a secondary contribution by precipitation of the warmest quarter (BIO18) (Table 2), a tertiary contribution by temperature annual range (BIO7) (Table 2), and lower contributions by the other variables. In general, temperature and precipitation most strongly influence the *D. minowai* distribution, whereas precipitation is the main influencer of *M. onukii.* As revealed in the MaxEnt response curves, the presence probability of *D. minowai* first increases and then decreases with increasing temperature annual range (BIO7) and increasing precipitation of the driest month (BIO14) (Figure 2). The presence probability of *M. onukii* follows the same pattern as *D. minowai* with respect to annual precipitation (BIO12), but initially increases and thereafter fluctuates before peaking as the precipitation of the warmest quarter (BIO18) increases (Figure 2).

### 3.2. Suitable Habitats and Extrapolation Risk

**Figure 3:**
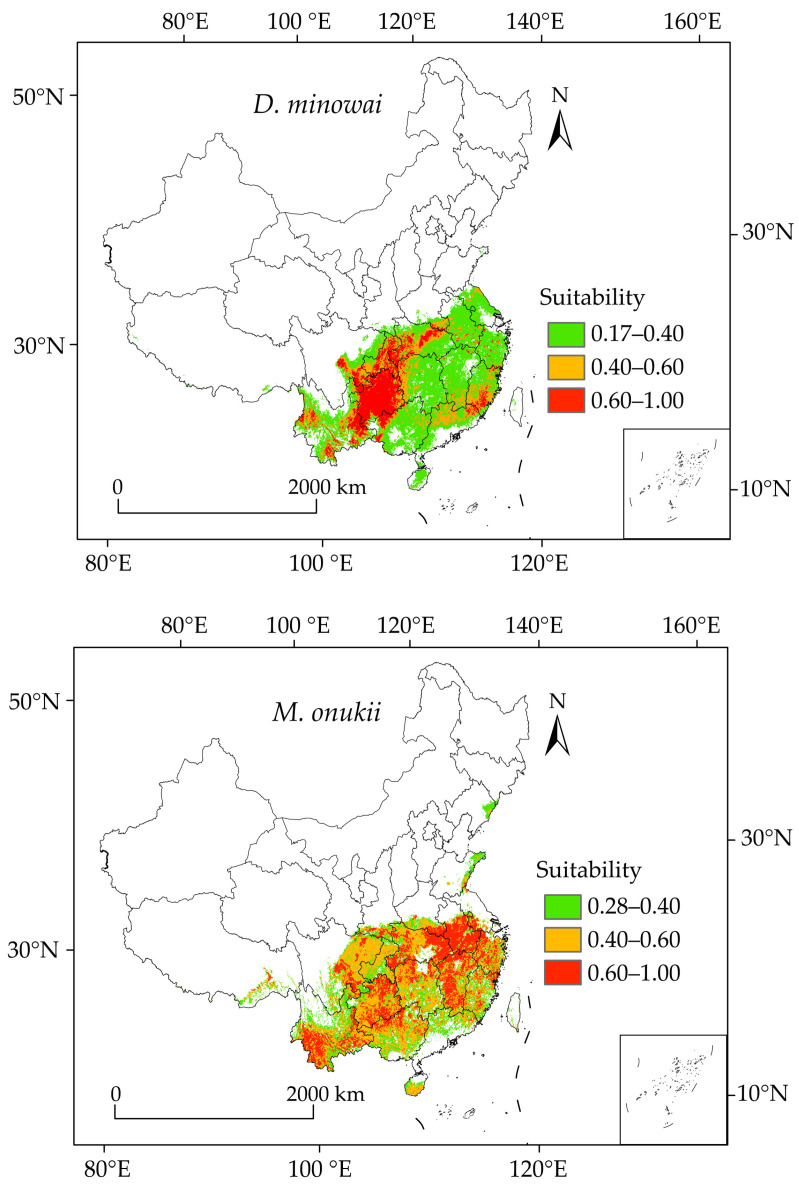
Suitable habitat patterns for *D. minowai* and *M. onukii* under current conditions in China.

**Figure 4:**
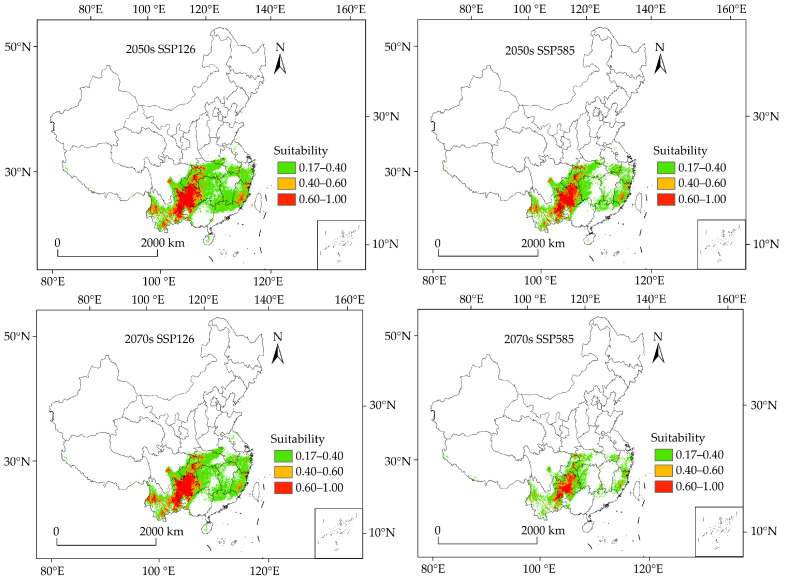
Suitable habitat patterns for *D. minowai* under future conditions in China.

According to the MaxEnt model under the current conditions, the suitable habitats of *D. minowai* are primarily distributed in provinces in central and southern China (Figure 3), concentrated in Yunnan, Guizhou, Chongqing, Guangxi, Hunan, Hubei, Jiangxi, Fujian, Zhejiang, and Anhui. Under future conditions, the suitable habitats for *D. minowai* are projected to shrink from those of the current conditions (Figure 4, Table 3). Moreover, the projected reduction in suitable habitat is most pronounced under the 2070s SSP585 climate scenario (Table 3), particularly in Guangxi, Hunan, Hubei, Jiangxi, Guangdong, Zhejiang, Jiangsu, and Anhui (Figure 4).

**Figure 5:**
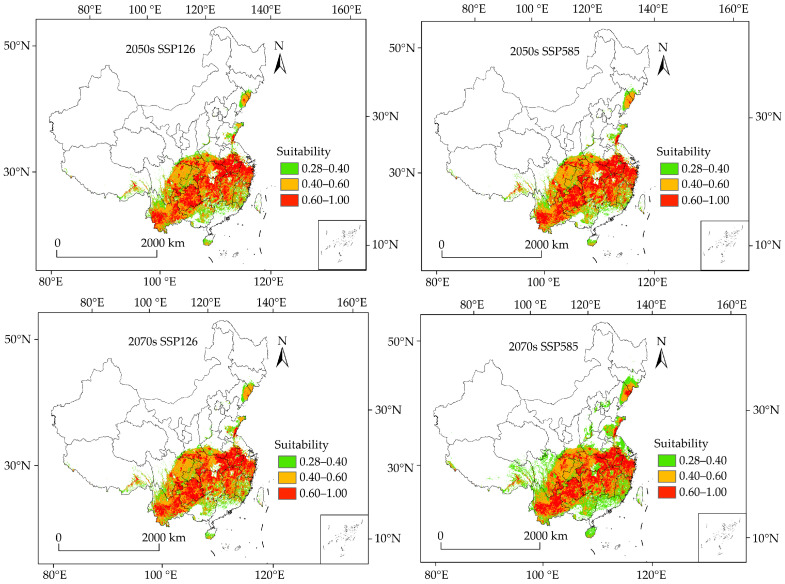
Suitable habitat patterns of *M. onukii* under future conditions in China.

In the present-time MaxEnt results, the geographical extent of the core suitable habitats of *M. onukii* aligns with that of *D. minowai* and spans the central and southern regions (Figure 3). The model also predicts a small proportion of suitable habitats in the Shandong and Liaoning regions in northern China (Figure 3). Under future conditions, the predicted suitable habitats of *M. onukii* are expected to expand (Figure 5). Expansion is maximized in the 2070s SSP585 climate scenario (Table 3), with notable increases in Xizang, Sichuan, Jiangsu, Shandong, and Liaoning (Figure 5).

**Figure 6:**
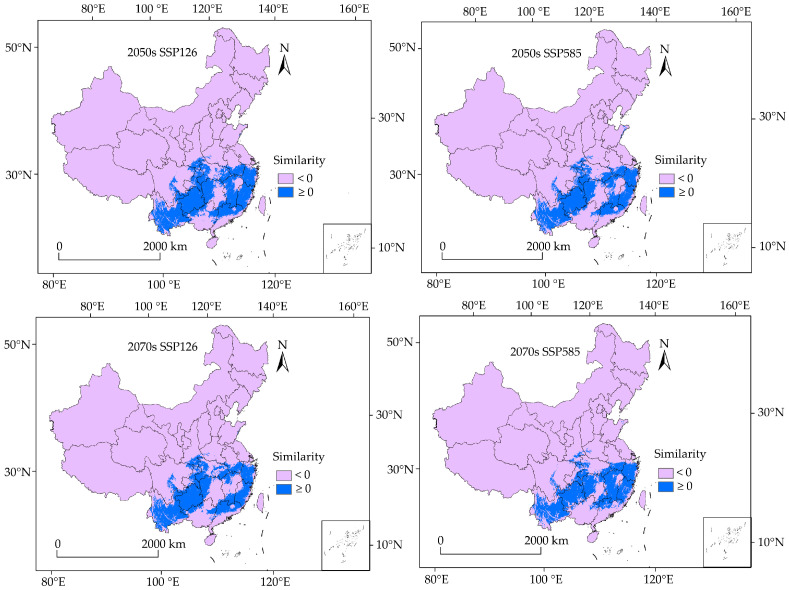
Extrapolation risk of the MaxEnt model for *D. minowai* based on multivariate environmental-similarity surfaces. Regions with similarity values ≥ 0 indicate no exploration risk, and regions with similarity values < 0 indicate an extrapolation risk.

**Figure 7:**
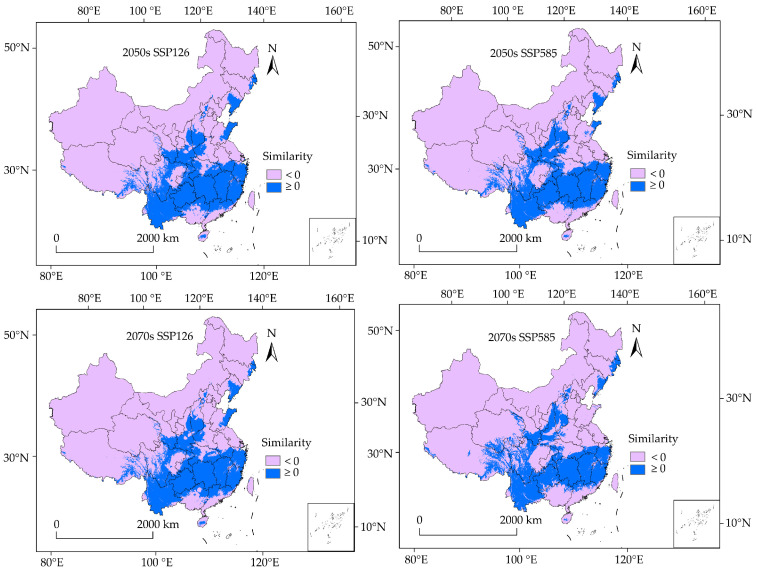
Extrapolation risk of the MaxEnt model for *M. onuki* based on multivariate environmental-similarity surfaces. Regions with similarity values ≥ 0 indicate no extrapolation risk, and regions with similarity values < 0 indicate an extrapolation risk.

Future habitat projections of both pests in the MaxEnt model reveal some extrapolation risks (Figure 6 and Figure 7). Distinct risk areas are observed in the eastern part of Hunan under the 2050s SSP126 scenario for *D. minowai* (Figure 6) and in the eastern part of Sichuan and the southern part of Guangxi in all future scenarios for *M. onukii* (Figure 7).

### 3.3. Overlap and Nonoverlap Patterns

**Figure 8:**
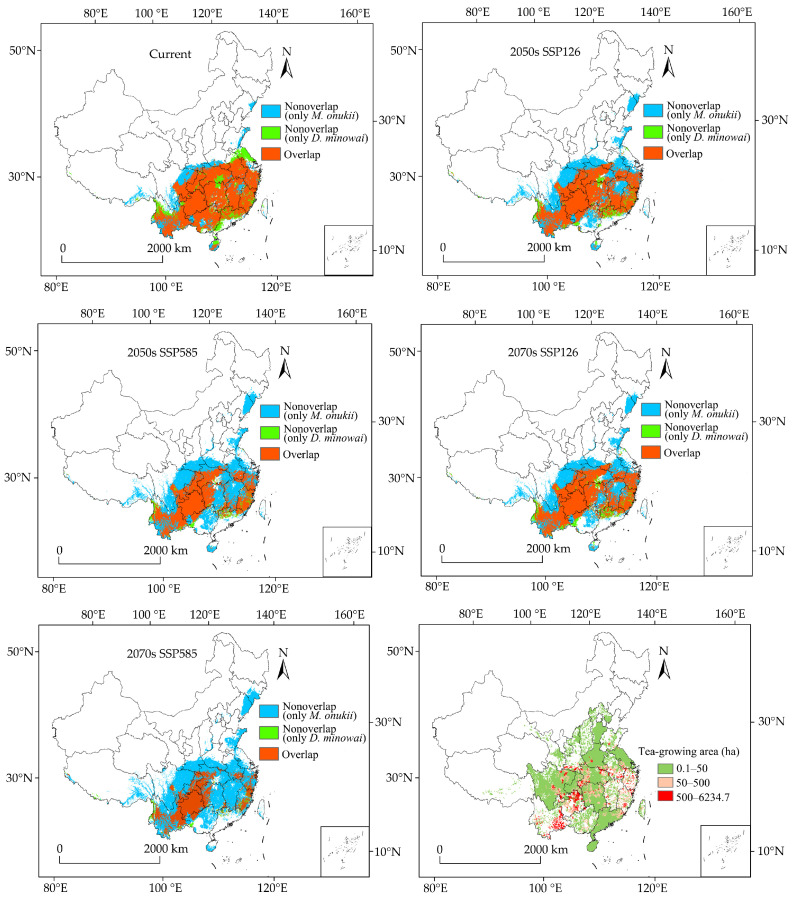
Overlap and nonoverlap areas under different conditions as well as in tea-growing areas in China.

The spatial-distribution patterns of the current and future overlap and nonoverlap areas were identified by overlapping the suitable habitats of *D. minowai* and *M. onukii*, (Figure 8). Under the current conditions, the overlap areas are larger than the nonoverlap areas (Table 3), and a substantial number of overlap areas are predicted in several provinces (Figure 8), including Guizhou, Yunnan, Chongqing, Hunan, Guangxi, Hubei, Jiangxi, Fujian, Zhejiang, and Anhui. The overlap areas are projected to decrease in future (Table 3), revealing reductions in the co-infestation zones of the two pests. Under the 2070s SSP585 climate scenario in particular, overlap areas are mainly distributed through Yunnan, Guizhou, Chongqing, Hunan, Fujian, and Zhejiang (Figure 8). Furthermore, the number of nonoverlap areas in the future can increase compared with the present period (Table 3).

## 4. Discussion

Infestation of tea trees by *D. minowai* and *M. onukii* has substantially reduced tea production in China. Understanding the geographical distribution patterns of the two pests is crucial for their management and control. To this end, we constructed a MaxEnt model that predicts the current and future suitable habitats of the two pests in China and thereby identifies the overlap areas. To our knowledge, we present the first such assessment for *D. minowai* and the second for *M. onukii*. The MaxEnt model fitted by Jiang et al. predicted a concentration of suitable *M. onukii* habitats in the central and southern regions of China under both current and future conditions [14], aligning with our prediction. Nevertheless, several major discrepancies appeared between the results of the two models. Jiang et al. predicted that the entire Guangxi, Guangdong, Hainan, and Taiwan regions are suitable for *M. onukii* [14], whereas our model predicts suitable *M. onukii* habitats only in restricted portions of these provinces. Moreover, Jiang et al. predicted numerous environmentally suitable habitats in Shanxi and Hebei [14], which were not identified in this study. These prediction discrepancies can be largely attributed to differences in the constructed model parameters. Specifically, the MaxEnt model of Jiang et al. used the default feature combination and regularization multiplier value [14]. As both parameters are related to model complexity, the default settings may lead to inaccurate predictions [38,39,40] and the degradation of model performance [41,42,43]. More diverse combinations of feature types, which are mathematical transformations of environmental variables, yield a more complex model construction [40]. By default, MaxEnt decides the used feature types based on the number of distribution records [44]. Model complexity is reduced through regularization, and the default value is determined by the performance on a series of taxonomic groups; however, it may be suboptimal when the number of species is small [44]. To obtain a reliable MaxEnt modeling framework, the feature combinations and regularization multiplier values must be appropriately selected based on specific evaluation metrics (e.g., the AICc) [32], as done in our study. Therefore, our model may predict more accurate *M. onukii* habitats than Jiang et al.’s model [14].

As empirically clarified in numerous species distribution models, different types of variables differently contribute to macro-scale insect pest distributions, with climate variables often playing a dominant role [45,46,47]. Consistent with this consensus, our MaxEnt model identified temperature variables and precipitation as the main factors determining the distribution of *D. minowai* and precipitation as the main influencer of *M. onukii* distribution. The response curves indicate that overall the occurrence of *D. minowai* is facilitated and inhibited at lower and higher temperature annual ranges, respectively (Figure 2). As the annual temperature range reflects the fluctuation magnitude of extreme temperatures, *D. minowai* will more likely be distributed in areas with smaller thermal fluctuations, namely, central and southern China (Figure 3). By contrast, suitable habitats were not present in the northern region with large fluctuations. Lower thermal fluctuations may reduce temperature stress, facilitating the establishment and maintenance of the *D. minowai* population. Both pests were detected only where the precipitation exceeds a certain threshold (Figure 2), possibly because such regions meet the minimum water requirements for pest growth and development. While *M. onukii* can cope with continuously increasing precipitation, *D. minowa* disappears in regions of excessive precipitation. This observation may be related to the smaller size of *D. minowa* (0.80–0.90 mm for female adults; 0.60–0.65 mm for male adults) [11] compared to *M. onukii*. As smaller insects are more easily washed away during heavy rainfall [48], the *D. minowai* population may not easily establish in such regions.

Our MaxEnt model predicts different spatial pattern changes in the distributions of the two pests in the context of future climate change. In particular, the suitable habitat of *D. minowai* is projected to decrease while that of *M. onukii* increases. Both response patterns have been reported in habitat predictions of other insect pests, such as *Bemisia tabaci* [49], *Lissorhoptrus oryzophilus* [50], and *Anarsia lineatella* [51]. These findings highlight species-specific responses to climate change. We used different environmental datasets and model parameters for *D. minowa* and *M. onukii*, which may result in different predicted responses of the two pests to climate change. Consequently, comparisons of model predictions may warrant cautious interpretation. Our prediction results also revealed more prominent changes in the suitable habitats of both pests under the 2070s SSP585 scenario than under the other future scenarios, implying that spatial restructuring of the *D. minowai* and *M. onuki* distribution patterns intensifies with increasing climate-change severity.

*D. minowai* and *M. onukii* can be detected at the same location [24], indicating that both pest populations can survive in similar ecological environments. Supporting this finding, our model identified spatial overlap of the suitable habitats of both pest species at the current time, which is projected to reduce under future climate change. This shrinkage can be attributed to a future decrease in suitable habitats of *D. minowai*. The overlapping areas represent the co-infestation zones of *D. minowai* and *M. onukii*, where the damage to tea trees is presumably higher than in regions inhabited by either pest species alone. By identifying overlap areas in the present and future periods, we can improve the precision and efficiency of managing the two pests. Such areas should be prioritized for management measures implemented by farmers and plant protection organizations. A wide body of resources, including professionals, funding, and pesticides, should be primarily allocated to these areas. The areas prioritized for management will require adjustment as the distribution range of overlap areas changes in future. For instance, overlap areas cover almost the entire territory of Jiangxi Province under the current conditions, but very few overlap areas are projected under the 2070s SSP585 scenario. Such areas will cease to require priority management under this scenario. In contrast, the present overlap areas in some provinces (e.g., Guizhou) will persist in future, suggesting a requirement for long-term management and control. More importantly, many provinces with large tea-growing areas (such as Yunnan, Guizhou, Hunan, and Fujian) are predicted as persistent overlap areas. To avoid serious and persistent infestation risks to tea trees, prioritized management is urgently required in overlap areas.

Identifying the extrapolation risk is crucial for assessing the prediction reliability of a species distribution model. Extrapolation is a common problem in model projections under novel environmental conditions [52,53,54] and was also detected in this study. Given their low reliability, the predicted suitable habitats with extrapolation risk for *D. minowai* and *M. onukii* should be interpreted with caution. In addition, the observed degree of extrapolation should be evaluated using other methods, such as Mobility-Oriented Parity [55] and Shape [56], in subsequent studies.

## 5. Conclusions

We constructed a MaxEnt model of the tea-tree pests *D. minowai* and *M. onukii* in China and optimized it by minimizing the AICc. Using the model, we then predicted the suitable habitats and overlap areas under both current and future climate conditions. The suitable habitats of *D. minowai* and *M. onukii* are concentrated in the central and southern provinces of China at present and are predicted to shrink and expand, respectively, in future. This change pattern is most severe under the most extreme climate-change scenario (the 2070s SSP585 scenario). The model results also highlight the urgency of designating overlap areas as priority management zones. Besides assisting our understanding of how future environmental changes will affect the distribution of *D. minowai* and *M. onukii*, our findings provide evidence-based management of these pests, which can be implemented by farmers and plant protection organizations. Overall, this study is anticipated to facilitate the development of China’s tea industry and agricultural economy.

## Figures and Tables

**Table 1:** Evaluation of the optimal MaxEnt for *D. minowai* and *M. onukii*.

Species	Feature Combination	Regularization Multiplier	AICc	AUC	pAUC Ratio
*D. minowai*	QHPT	1.8	1548.70	0.94	1.85
*M. onukii*	HPT	1.2	5758.39	0.89	1.66

**Table 2:** Contributions of different environmental variables to *D. minowai* and *M. onukii* distributions, obtained through a permutation analysis.

Environmental Variables	*D. minowai*	*M. onukii*
Temperature annual range (BIO7)	46.0	16.7
Precipitation of driest month (BIO14)	45.3	–
Annual precipitation (BIO12)	–	21.5
Precipitation of warmest quarter (BIO18)	0.6	21.4
Mean temperature of wettest quarter (BIO8)	3.1	16.0
Mean diurnal range (BIO2)	1.2	3.0
Mean temperature of driest quarter (BIO9)	1.0	–
Tea-growing area	1.0	9.0
Precipitation seasonality (BIO15)	–	2.1
Terrain ruggedness	1.7	–
Slope	–	10.3

“–” this variable was not included in the MaxEnt model.

**Table 3:** Suitable habitat, overlap, and nonoverlap areas under different climate conditions (×10^5^ km^2^).

Type	Current	2050s	2050s	2070s	2070s
		SSP126	SSP585	SSP126	SSP585
*D. minowai*	18.48	13.87 (−29.94%)	10.58 (−42.74%)	12.99 (−29.70%)	7.04 (−61.90%)
*M. onukii*	19.62	21.26 (+8.35%)	21.86 (+11.41%)	21.20 (+8.05%)	28.18 (+43.62%)
Overlap areas	15.74	12.54 (−20.33%)	9.79 (−37.80%)	11.68 (−25.79%)	6.53 (−58.51%)
Nonoverlap areas	6.63	10.04 (+51.43%)	12.86 (+93.96%)	10.82 (+63.19%)	16.34 (+146.45%)

## Data Availability

The original data presented are included in the Supplementary Materials.

## Supplementary Materials

The following supporting information can be downloaded at https://www.mdpi.com/article/10.3390/insects17070740/s1, Table S1: The distribution records of two pests.

## Abbreviations

The following abbreviations are used in this manuscript:

*D. minowai*	*Dendrothrips minowai*
*M. onukii*	*Matsumurasca (Matsumurasca) onukii*
AICc	Corrected Akaike information criterion
AUC	Area under the receiver operating characteristic curve
pAUC	Partial AUC
CQ	Chongqing
FJ	Fujian
HB	Hubei
HN	Hunan
GX	Guangxi
GZ	Guizhou
JX	Jiangxi
SX	Shaanxi
