# Supplementary material for: Suitable Habitats of Two Tea Pests for Management Guidance in China Under Climate Change
Source: Insects. 2026 Jul 20;17(7):740. doi: 10.3390/insects17070740 (PMC13411802; doi:10.3390/insects17070740)
Supplement: Supplementary file 1 [file insects-17-00740-s001.zip › insects-4414840-supplementary.pdf]

Table S1 The distribution records of two pests

Species	E	N
Dendrothrips minowai	100.08	23.88
Dendrothrips minowai	107.46	27.77
Dendrothrips minowai	120.58	29.98
Dendrothrips minowai	120.15	30.28
Dendrothrips minowai	118.03	27.77
Dendrothrips minowai	106.67	26.41
Dendrothrips minowai	108.94	33.16
Dendrothrips minowai	117.90	25.53
Dendrothrips minowai	110.34	31.07
Dendrothrips minowai	107.88	27.24
Dendrothrips minowai	119.57	27.21
Dendrothrips minowai	107.71	27.96
Dendrothrips minowai	104.51	28.45
Dendrothrips minowai	114.09	32.15
Dendrothrips minowai	105.39	26.15
Dendrothrips minowai	105.34	26.08
Dendrothrips minowai	115.94	28.56
Dendrothrips minowai	108.84	27.71
Dendrothrips minowai	105.21	25.85
Dendrothrips minowai	109.29	22.43
Dendrothrips minowai	97.79	24.20
Dendrothrips minowai	108.22	27.53
Dendrothrips minowai	121.54	29.87
Dendrothrips minowai	104.82	26.60
Dendrothrips minowai	116.02	28.37
Dendrothrips minowai	107.49	27.72
Dendrothrips minowai	106.42	26.35
Dendrothrips minowai	120.83	29.75
Dendrothrips minowai	119.90	30.40
Dendrothrips minowai	120.03	30.13
Dendrothrips minowai	107.59	28.63
Dendrothrips minowai	104.78	25.40
Dendrothrips minowai	104.99	25.12
Dendrothrips minowai	107.24	25.57
Dendrothrips minowai	107.55	27.73
Dendrothrips minowai	106.42	26.15
Dendrothrips minowai	106.45	26.02
Dendrothrips minowai	103.41	29.53
Dendrothrips minowai	109.53	18.73
Dendrothrips minowai	111.30	23.56
Dendrothrips minowai	113.39	24.30
Dendrothrips minowai	108.26	27.77

Dendrothrips minowai	100.96	22.75
Dendrothrips minowai	100.43	21.99
Dendrothrips minowai	106.65	26.50
Dendrothrips minowai	107.09	26.22
Dendrothrips minowai	108.16	27.61
Dendrothrips minowai	107.10	27.01
Dendrothrips minowai	105.54	26.20
Dendrothrips minowai	107.59	28.11
Dendrothrips minowai	107.57	25.98
Dendrothrips minowai	106.97	26.18
Dendrothrips minowai	108.70	27.65
Dendrothrips minowai	105.20	27.01
Dendrothrips minowai	105.08	25.79
Dendrothrips minowai	105.15	25.79
Dendrothrips minowai	108.11	27.98
Dendrothrips minowai	107.57	26.98
Dendrothrips minowai	107.61	28.86
Dendrothrips minowai	104.83	26.30
Dendrothrips minowai	107.37	28.26
Dendrothrips minowai	105.97	26.03
Dendrothrips minowai	108.08	26.38
Dendrothrips minowai	107.52	26.26
Dendrothrips minowai	104.78	24.96
Dendrothrips minowai	107.32	25.82
Dendrothrips minowai	105.68	27.22
Dendrothrips minowai	106.11	27.04
Dendrothrips minowai	105.32	27.40
Dendrothrips minowai	104.28	26.86
Matsumurasca (Matsumurasca) onukii	119.57	27.20
Matsumurasca (Matsumurasca) onukii	109.66	18.88
Matsumurasca (Matsumurasca) onukii	118.15	29.50
Matsumurasca (Matsumurasca) onukii	104.95	25.80
Matsumurasca (Matsumurasca) onukii	117.89	25.59
Matsumurasca (Matsumurasca) onukii	108.34	27.73
Matsumurasca (Matsumurasca) onukii	120.12	30.26
Matsumurasca (Matsumurasca) onukii	109.00	28.46
Matsumurasca (Matsumurasca) onukii	114.90	31.54
Matsumurasca (Matsumurasca) onukii	110.42	29.81
Matsumurasca (Matsumurasca) onukii	107.88	27.24
Matsumurasca (Matsumurasca) onukii	116.01	28.34
Matsumurasca (Matsumurasca) onukii	117.17	23.73
Matsumurasca (Matsumurasca) onukii	114.26	26.72
Matsumurasca (Matsumurasca) onukii	103.74	29.92
Matsumurasca (Matsumurasca) onukii	116.67	23.48

Matsumurasca (Matsumurasca) onukii	119.63	31.26
Matsumurasca (Matsumurasca) onukii	115.94	28.56
Matsumurasca (Matsumurasca) onukii	114.52	26.33
Matsumurasca (Matsumurasca) onukii	106.87	32.75
Matsumurasca (Matsumurasca) onukii	106.12	32.73
Matsumurasca (Matsumurasca) onukii	119.48	28.45
Matsumurasca (Matsumurasca) onukii	107.71	27.97
Matsumurasca (Matsumurasca) onukii	119.46	35.44
Matsumurasca (Matsumurasca) onukii	121.59	37.40
Matsumurasca (Matsumurasca) onukii	105.61	29.42
Matsumurasca (Matsumurasca) onukii	106.54	29.42
Matsumurasca (Matsumurasca) onukii	108.40	30.82
Matsumurasca (Matsumurasca) onukii	119.91	28.46
Matsumurasca (Matsumurasca) onukii	119.63	27.99
Matsumurasca (Matsumurasca) onukii	119.14	28.09
Matsumurasca (Matsumurasca) onukii	116.23	30.79
Matsumurasca (Matsumurasca) onukii	116.30	30.33
Matsumurasca (Matsumurasca) onukii	116.57	30.52
Matsumurasca (Matsumurasca) onukii	117.71	29.87
Matsumurasca (Matsumurasca) onukii	115.92	31.76
Matsumurasca (Matsumurasca) onukii	117.02	30.00
Matsumurasca (Matsumurasca) onukii	111.48	28.94
Matsumurasca (Matsumurasca) onukii	110.39	28.49
Matsumurasca (Matsumurasca) onukii	111.13	29.44
Matsumurasca (Matsumurasca) onukii	111.21	28.39
Matsumurasca (Matsumurasca) onukii	111.37	29.60
Matsumurasca (Matsumurasca) onukii	113.58	28.72
Matsumurasca (Matsumurasca) onukii	117.35	24.53
Matsumurasca (Matsumurasca) onukii	116.95	24.28
Matsumurasca (Matsumurasca) onukii	118.29	25.34
Matsumurasca (Matsumurasca) onukii	119.65	27.49
Matsumurasca (Matsumurasca) onukii	118.24	25.54
Matsumurasca (Matsumurasca) onukii	118.18	25.07
Matsumurasca (Matsumurasca) onukii	119.45	27.17
Matsumurasca (Matsumurasca) onukii	114.50	32.22
Matsumurasca (Matsumurasca) onukii	114.54	29.04
Matsumurasca (Matsumurasca) onukii	117.21	29.36
Matsumurasca (Matsumurasca) onukii	109.27	32.40
Matsumurasca (Matsumurasca) onukii	120.28	31.53
Matsumurasca (Matsumurasca) onukii	120.63	31.28
Matsumurasca (Matsumurasca) onukii	119.43	32.01
Matsumurasca (Matsumurasca) onukii	110.18	24.15
Matsumurasca (Matsumurasca) onukii	110.80	24.17
Matsumurasca (Matsumurasca) onukii	109.29	22.43

Matsumurasca (Matsumurasca) onukii	109.65	25.93
Matsumurasca (Matsumurasca) onukii	109.21	27.52
Matsumurasca (Matsumurasca) onukii	107.44	28.56
Matsumurasca (Matsumurasca) onukii	105.96	26.26
Matsumurasca (Matsumurasca) onukii	104.15	28.67
Matsumurasca (Matsumurasca) onukii	105.06	28.74
Matsumurasca (Matsumurasca) onukii	103.34	30.25
Matsumurasca (Matsumurasca) onukii	103.04	30.01
Matsumurasca (Matsumurasca) onukii	117.84	25.71
Matsumurasca (Matsumurasca) onukii	106.67	26.41
Matsumurasca (Matsumurasca) onukii	118.93	25.88
Matsumurasca (Matsumurasca) onukii	107.32	25.84
Matsumurasca (Matsumurasca) onukii	120.56	27.68
Matsumurasca (Matsumurasca) onukii	118.63	31.24
Matsumurasca (Matsumurasca) onukii	111.65	32.28
Matsumurasca (Matsumurasca) onukii	103.88	29.94
Matsumurasca (Matsumurasca) onukii	117.07	30.56
Matsumurasca (Matsumurasca) onukii	119.33	27.11
Matsumurasca (Matsumurasca) onukii	119.32	26.09
Matsumurasca (Matsumurasca) onukii	119.27	26.05
Matsumurasca (Matsumurasca) onukii	116.69	30.49
Matsumurasca (Matsumurasca) onukii	106.74	26.34
Matsumurasca (Matsumurasca) onukii	114.12	31.57
Matsumurasca (Matsumurasca) onukii	119.68	30.65
Matsumurasca (Matsumurasca) onukii	107.74	28.11
Matsumurasca (Matsumurasca) onukii	107.07	26.22
Matsumurasca (Matsumurasca) onukii	118.81	27.47
Matsumurasca (Matsumurasca) onukii	107.51	26.27
Matsumurasca (Matsumurasca) onukii	100.02	24.53
Matsumurasca (Matsumurasca) onukii	119.09	31.07
Matsumurasca (Matsumurasca) onukii	109.68	25.76
Matsumurasca (Matsumurasca) onukii	104.47	28.68
Matsumurasca (Matsumurasca) onukii	115.73	31.59
Matsumurasca (Matsumurasca) onukii	119.66	27.08
Matsumurasca (Matsumurasca) onukii	107.76	32.99
Matsumurasca (Matsumurasca) onukii	119.62	29.00
Matsumurasca (Matsumurasca) onukii	110.73	24.07
Matsumurasca (Matsumurasca) onukii	100.54	22.02
Matsumurasca (Matsumurasca) onukii	120.10	30.21
Matsumurasca (Matsumurasca) onukii	114.36	29.93
Matsumurasca (Matsumurasca) onukii	117.62	25.87
Matsumurasca (Matsumurasca) onukii	114.32	30.39
Matsumurasca (Matsumurasca) onukii	118.71	30.98
Matsumurasca (Matsumurasca) onukii	113.84	32.18

Matsumurasca (Matsumurasca) onukii	111.03	28.36
Matsumurasca (Matsumurasca) onukii	116.70	23.90
Matsumurasca (Matsumurasca) onukii	119.93	27.26
Matsumurasca (Matsumurasca) onukii	114.29	29.86
Matsumurasca (Matsumurasca) onukii	119.20	26.10
Matsumurasca (Matsumurasca) onukii	119.82	28.89
Matsumurasca (Matsumurasca) onukii	110.34	25.28
Matsumurasca (Matsumurasca) onukii	109.92	19.05
Matsumurasca (Matsumurasca) onukii	103.69	29.79
Matsumurasca (Matsumurasca) onukii	107.48	27.77
Matsumurasca (Matsumurasca) onukii	105.92	29.40
Matsumurasca (Matsumurasca) onukii	101.81	24.57
Matsumurasca (Matsumurasca) onukii	100.96	22.75
Matsumurasca (Matsumurasca) onukii	101.61	23.10
Matsumurasca (Matsumurasca) onukii	100.43	21.98
Matsumurasca (Matsumurasca) onukii	100.03	23.85
Matsumurasca (Matsumurasca) onukii	100.83	24.45
Matsumurasca (Matsumurasca) onukii	114.06	32.09
Matsumurasca (Matsumurasca) onukii	119.26	35.29
Matsumurasca (Matsumurasca) onukii	117.24	36.17
Matsumurasca (Matsumurasca) onukii	115.72	28.81
Matsumurasca (Matsumurasca) onukii	114.37	28.52
Matsumurasca (Matsumurasca) onukii	106.66	24.50
Matsumurasca (Matsumurasca) onukii	111.16	28.64
Matsumurasca (Matsumurasca) onukii	117.90	27.64
Matsumurasca (Matsumurasca) onukii	98.67	24.92
Matsumurasca (Matsumurasca) onukii	100.50	21.79
Matsumurasca (Matsumurasca) onukii	102.27	24.14
Matsumurasca (Matsumurasca) onukii	95.35	29.19
Matsumurasca (Matsumurasca) onukii	94.97	30.16
Matsumurasca (Matsumurasca) onukii	94.81	30.24
Matsumurasca (Matsumurasca) onukii	103.38	30.16
Matsumurasca (Matsumurasca) onukii	104.45	31.81
Matsumurasca (Matsumurasca) onukii	105.15	25.78
Matsumurasca (Matsumurasca) onukii	107.47	26.35
Matsumurasca (Matsumurasca) onukii	107.70	28.02
Matsumurasca (Matsumurasca) onukii	108.41	31.36
Matsumurasca (Matsumurasca) onukii	109.50	30.06
Matsumurasca (Matsumurasca) onukii	110.58	32.47
Matsumurasca (Matsumurasca) onukii	114.50	30.99
Matsumurasca (Matsumurasca) onukii	109.87	28.62
Matsumurasca (Matsumurasca) onukii	110.48	27.05
Matsumurasca (Matsumurasca) onukii	113.26	28.30
Matsumurasca (Matsumurasca) onukii	116.22	31.39

Matsumurasca (Matsumurasca) onukii	117.51	29.84
Matsumurasca (Matsumurasca) onukii	117.87	32.65
Matsumurasca (Matsumurasca) onukii	118.00	30.32
Matsumurasca (Matsumurasca) onukii	119.31	34.66
Matsumurasca (Matsumurasca) onukii	117.20	31.93
Matsumurasca (Matsumurasca) onukii	119.14	30.98
Matsumurasca (Matsumurasca) onukii	118.21	30.25
Matsumurasca (Matsumurasca) onukii	119.89	30.40
Matsumurasca (Matsumurasca) onukii	120.15	31.25
Matsumurasca (Matsumurasca) onukii	113.08	28.20
Matsumurasca (Matsumurasca) onukii	113.33	28.46
Matsumurasca (Matsumurasca) onukii	113.38	24.30
Matsumurasca (Matsumurasca) onukii	109.24	19.15
Matsumurasca (Matsumurasca) onukii	116.82	29.48
Matsumurasca (Matsumurasca) onukii	114.12	32.16
Matsumurasca (Matsumurasca) onukii	100.88	22.74
Matsumurasca (Matsumurasca) onukii	101.23	21.99
Matsumurasca (Matsumurasca) onukii	100.43	22.68
Matsumurasca (Matsumurasca) onukii	107.23	27.44
Matsumurasca (Matsumurasca) onukii	106.49	29.22
Matsumurasca (Matsumurasca) onukii	106.71	29.51
Matsumurasca (Matsumurasca) onukii	100.58	23.73
Matsumurasca (Matsumurasca) onukii	116.05	30.06
Matsumurasca (Matsumurasca) onukii	118.86	35.12
Matsumurasca (Matsumurasca) onukii	107.99	32.25
Matsumurasca (Matsumurasca) onukii	100.18	22.09
Matsumurasca (Matsumurasca) onukii	95.77	30.15
Matsumurasca (Matsumurasca) onukii	117.50	30.64
Matsumurasca (Matsumurasca) onukii	116.94	31.46
Matsumurasca (Matsumurasca) onukii	119.65	30.73
Matsumurasca (Matsumurasca) onukii	120.04	30.18
Matsumurasca (Matsumurasca) onukii	116.02	29.67
Matsumurasca (Matsumurasca) onukii	113.91	31.98
Matsumurasca (Matsumurasca) onukii	113.89	32.05
Matsumurasca (Matsumurasca) onukii	118.14	32.28
Matsumurasca (Matsumurasca) onukii	119.41	31.79
Matsumurasca (Matsumurasca) onukii	118.94	30.96
Matsumurasca (Matsumurasca) onukii	107.83	32.41
Matsumurasca (Matsumurasca) onukii	99.86	23.66
Matsumurasca (Matsumurasca) onukii	119.64	32.42
Matsumurasca (Matsumurasca) onukii	106.58	32.96
Matsumurasca (Matsumurasca) onukii	118.97	35.02
Matsumurasca (Matsumurasca) onukii	113.85	32.12
Matsumurasca (Matsumurasca) onukii	103.21	30.21

Matsumurasca (Matsumurasca) onukii	99.91	23.62
Matsumurasca (Matsumurasca) onukii	116.72	31.27
Matsumurasca (Matsumurasca) onukii	116.15	31.61
Matsumurasca (Matsumurasca) onukii	115.92	30.86
Matsumurasca (Matsumurasca) onukii	100.07	24.47
Matsumurasca (Matsumurasca) onukii	100.39	23.67
Matsumurasca (Matsumurasca) onukii	115.46	30.38
Matsumurasca (Matsumurasca) onukii	103.39	30.95
Matsumurasca (Matsumurasca) onukii	117.63	31.85
Matsumurasca (Matsumurasca) onukii	95.67	30.05
Matsumurasca (Matsumurasca) onukii	119.15	35.28
Matsumurasca (Matsumurasca) onukii	100.04	23.71
Matsumurasca (Matsumurasca) onukii	117.58	31.34
Matsumurasca (Matsumurasca) onukii	108.58	32.61
Matsumurasca (Matsumurasca) onukii	106.67	32.94
Matsumurasca (Matsumurasca) onukii	119.66	31.98
Matsumurasca (Matsumurasca) onukii	116.91	31.05
Matsumurasca (Matsumurasca) onukii	118.66	31.71
Matsumurasca (Matsumurasca) onukii	99.58	24.67
Matsumurasca (Matsumurasca) onukii	119.18	32.12
Matsumurasca (Matsumurasca) onukii	108.11	32.60
Matsumurasca (Matsumurasca) onukii	115.72	30.75
Matsumurasca (Matsumurasca) onukii	99.63	24.75
Matsumurasca (Matsumurasca) onukii	118.59	30.81
Matsumurasca (Matsumurasca) onukii	117.75	29.84
Matsumurasca (Matsumurasca) onukii	95.33	29.33
Matsumurasca (Matsumurasca) onukii	117.08	31.31
Matsumurasca (Matsumurasca) onukii	106.65	33.04
Matsumurasca (Matsumurasca) onukii	116.78	31.18
Matsumurasca (Matsumurasca) onukii	91.75	27.82
Matsumurasca (Matsumurasca) onukii	108.66	32.58
Matsumurasca (Matsumurasca) onukii	107.88	32.67
Matsumurasca (Matsumurasca) onukii	120.49	24.12
Matsumurasca (Matsumurasca) onukii	109.88	18.79
Matsumurasca (Matsumurasca) onukii	110.00	18.83
Matsumurasca (Matsumurasca) onukii	101.25	21.94
Matsumurasca (Matsumurasca) onukii	100.09	23.89
Matsumurasca (Matsumurasca) onukii	99.93	24.58
Matsumurasca (Matsumurasca) onukii	112.92	24.93
Matsumurasca (Matsumurasca) onukii	99.16	25.12
Matsumurasca (Matsumurasca) onukii	113.02	25.77
Matsumurasca (Matsumurasca) onukii	112.86	27.23
Matsumurasca (Matsumurasca) onukii	117.33	27.54
Matsumurasca (Matsumurasca) onukii	117.63	27.73

Matsumurasca (Matsumurasca) onukii	117.68	27.75
Matsumurasca (Matsumurasca) onukii	118.03	27.76
Matsumurasca (Matsumurasca) onukii	110.46	29.37
Matsumurasca (Matsumurasca) onukii	103.35	29.56
Matsumurasca (Matsumurasca) onukii	102.14	29.64
Matsumurasca (Matsumurasca) onukii	117.53	30.02
Matsumurasca (Matsumurasca) onukii	114.08	31.81
Matsumurasca (Matsumurasca) onukii	105.24	32.75
Matsumurasca (Matsumurasca) onukii	121.72	41.61
Matsumurasca (Matsumurasca) onukii	117.60	42.51
